# Extracellular Interaction of *Bacillus thuringiensis*, ATP and Phage 0105phi7-2: A Potential New Anti-Bacterial Strategy

**DOI:** 10.3390/v15122409

**Published:** 2023-12-12

**Authors:** Samantha Ritter, Elena T. Wright, Philip Serwer

**Affiliations:** 1 Department of Microbiology, Immunology and Molecular Genetics, UT Health, San Antonio, TX 78229, USA; robertss1@livemail.uthscsa.edu; 2 Department of Biochemistry and Structural Biology, UT Health, San Antonio, TX 78229, USA; wrighte@uthscsa.edu

**Keywords:** ATP concentration gradients, ATP signaling, biofilms, chemotaxis, phage ride-hitching

## Abstract

The following hypothesis proposes non-diffusive, environmental bacteriophage (phage) motion. (1) Some phage-hosting, motile bacteria undergo chemotaxis down ATP concentration gradients to escape lysis-inducing conditions, such as phage infection. (2) Some phages respond by non-infective binding to the motile bacteria. (3) When the bacteria reach a lower ATP concentration, which is a condition that signals increased density of phage-susceptible bacteria, the phage converts, Trojan-horse-like, to productive binding and infection. This hypothesis was previously proposed for *Bacillus thuringiensis* siphophage 0105phi7-2. It is tested here and confirmed with the following observations. (1) *B. thuringiensis* is found, macroscopically, preferentially located at low ATP concentrations when propagated in-gel after inoculation in the center of an artificially generated ATP concentration gradient. (2) Inoculating phage 0105phi7-2 at the bacteria inoculation site, 2–3 h after inoculation of bacteria, results in cell lysing activity that moves with the bacteria, without a visible trail of lysis. Trojan-horse-like behavior is consistent with only biofilm-inhabiting phages because environmental selection for this behavior requires limited fluid flows. We propose using artificial ATP concentration gradients to instigate Trojan-horse-like phage behavior during phage therapy of bacterial biofilms.

## 1. Introduction

Extracellular phage activity is a component of both microbial evolution and biomedical phage uses, including uses for therapy of biofilm-associated bacterial infections. Biofilms, a location of some disease/problem-causing, often antibiotic-resistant bacteria, consist primarily of the bacterial cells embedded in a gelled matrix. The matrix is usually formed primarily by polysaccharides that the bacteria secrete [1,2,3,4,5]. In at least some cases, the early-stage matrix is enhanced by the presence of bacterial DNA [6,7]. ATP plays a role in signaling biofilm formation, as an extracellular molecule (eATP) that is intercepted by bacteria when bacterially damaged cells use eATP to initiate an anti-bacterial immune response [7,8].

Thus, this anti-bacterial eATP signal also initiates bacterial defensive activity that sometimes includes biofilm formation [7] and sometimes the opposite [8]. In addition to being a biofilm initiator, bacterial motility is usually, but not always, in the pathway of the formation of biofilms [9,10,11,12]. In this context, defensive, chemorepellent, ATP-induced chemotaxis has been seen [13] and subsequently discussed [14] for paramecia, however, not, to our knowledge, for motile bacteria. This raises the question of whether bacteria, in analogy with paramecia, use eATP as a chemorepellent. The danger signaling and possible chemorepellent effect of eATP on bacteria raise the further question of whether eATP has these functions when the anti-bacterial danger is a bacterial virus (bacteriophage or phage). In this context, our previous observations of the biological and physical properties of newly characterized siphophage 0105phi7-2 led to the following hypothesis [15]. 

(1) High ATP concentration ([ATP]) is a danger signal to bacteria because high [ATP] indicates the presence of a bacterial cell-damaging agent, e.g., a phage. (2) If motile, some bacteria undergo chemotaxis down an eATP gradient, the past evolutionary selection for this activity having been the avoidance of danger. (3) In response, some phages bind to motile bacteria and, while so doing, assume a non-infective state. A consequence is (anthropomorphically speaking) “ride-hitching”. (4) The bacteria drag the ride-hitching phages to a region of lower [ATP] and, when [ATP] drops enough, the phages become infective for the bacteria.

The idea of phage ride-hitching (also called hitchhiking) has also been presented in a different context: increasing the frequency of phage–host contacts (recent review: [16]). The selective advantage of (1)–(4) for the phage is that, after ride-hitching, the phage is, Trojan-horse-like, in the presence of an increased concentration of live, phage-susceptible bacteria.

The current study tests the following aspects of the above hypothesis. (1) The host of 0105phi7-2, *B. thuringiensis*, undergoes chemotaxis down a gradient of [ATP]. (2) Phage 0105phi7-2, in a non-infective state, accompanies the host down the gradient of [ATP] and then converts to an infective state.

## 2. Materials and Methods

### 2.1. Phage and Host

Phage 0105phi7-2 and its motile host, *Bacillus thuringiensis*, have been previously described [15]. Phage 0105phi7-2 was propagated in-gel and purified by procedure ending with rate zonal centrifugation in a sucrose density gradient, as previously described [15].

Liquid, stationary-phase bacterial cultures were obtained in the following medium: 10 g tryptone, 5 g KCl in 1 L of water (T broth) to which a 1000 dilution of 1.0 M CaCl_2_ was added after separate autoclaving. Bacteria used below had been propagated overnight at room temperature (22 ± 3 °C).

### 2.2. Gelled Media and Generation of an Extracellular [ATP] Gradient

For testing the effect of an extracellular [ATP] gradient, bacteria were propagated in a gel that was the top layer within a Petri dish that had two gelled layers. The bottom layer was gelled 1.0% Bacto agar in T broth. The top layer was gelled agarose (Seakem Gold, Lonza, Rockland, ME, USA) in medium used for propagating bacteria. The concentration of agarose is in the text. Seakem Gold agarose has relatively high gel strength [17], which assists its use at the relatively low concentration in the experiments below.

To generate an [ATP] gradient, the bottom agar layer was poured in two sections. The first and bottom section was poured with the Petri dish tilted so that the subsequent gel was no more than 1 mm high at the thin end and about 7 mm high at the thick end. This section had ATP added to the pre-gelled agar solution at 50 °C. The [ATP] is indicated in the text. The second section of the bottom agar layer was (1) roughly equal in volume to the first section, (2) added after gelation of the ATP-containing section for 30–40 min, and (3) poured with the Petri dish level. The result was two wedge-shaped components of the bottom layer agar gel, the bottom one with ATP, and the other without ATP.

After pouring the two gelled layers, the Petri dish was incubated, within one hour, as described below. During incubation, ATP diffusion generated an [ATP] gradient in both the bottom agar gel and the upper-layer agarose gel. In the images of a Petri dish, the high [ATP] side of the Petri dish is on the right. This [ATP] gradient-producing procedure has previously been used to generate concentration gradients of other compounds, including mutagens [18], anti-fungal compounds [19], and anti-bacterial compounds [20]. As a control for the effect of ATP without an [ATP] gradient, ATP was also included in the top agar wedge at the concentration used for the lower agar wedge.

### 2.3. Testing the Effects of an [ATP] Gradient on Bacteria and Phage

To test the effects of an [ATP] gradient on the positioning of motile bacteria, a two-section, [ATP] gradient-generating agar gel was overlayed with molten top layer 0.175% agarose. The top layer was allowed to gel for 30–40 min. Then, bacteria were placed in the center of the top layer gel by use of a glass micropipette containing ~20 μL of an overnight culture at a concentration of 1.6–2.0 × 10^8^ bacteria per mL, as seen by phase contrast light microscopy. The Petri dish was then incubated for 19.0 h. To determine where the bacteria were after incubation, visible-light scattering was observed and photographed. Control incubation with [ATP] the same in both agar wedges was performed for 24.0 h.

The effect of this procedure on the positioning of infective phages was determined by doing the following. Phages were added to the gel at the position of the previous addition of bacteria, after a time and in amount indicated in the text.

## 3. Results

### 3.1. Effects of an Extracellular [ATP] Gradient vs. [ATP] in the Lower Agar Wedge

**Figure 1:**
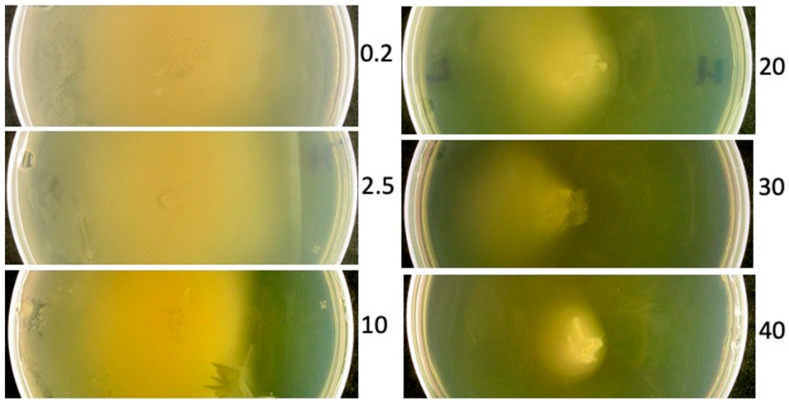
Response of *B. thuringiensis* to propagation in a 0.175% upper layer agarose gel exposed to an [ATP] gradient and incubated. Results are shown as a function of the [ATP] in the bottom wedge of the agar gel on which the upper layer agarose gel rests. The [ATP] (mM) is indicated at the right of each Petri plate. The right of each Petri plate is also the high [ATP] side; the low [ATP] side is opposite.

Testing of the above hypothesis, part (1) (i.e., chemotaxis), was performed by inoculating host cells in the center of a Petri dish-contained, growth medium-containing, upper, 0.175% agarose gel that had an artificially generated [ATP] gradient (Section 2.2 and Section 2.3). The Petri dish was then incubated. Bacteria moved within the gel and propagated during this time. Results were observed through visible-light scattering the next day. These results were obtained for lower agar wedge-[ATP] between 0.2 mM and 40 mM (Figure 1; the [ATP] in mM is indicated at the high [ATP] side of a gel, to the right).

**Figure 2:**
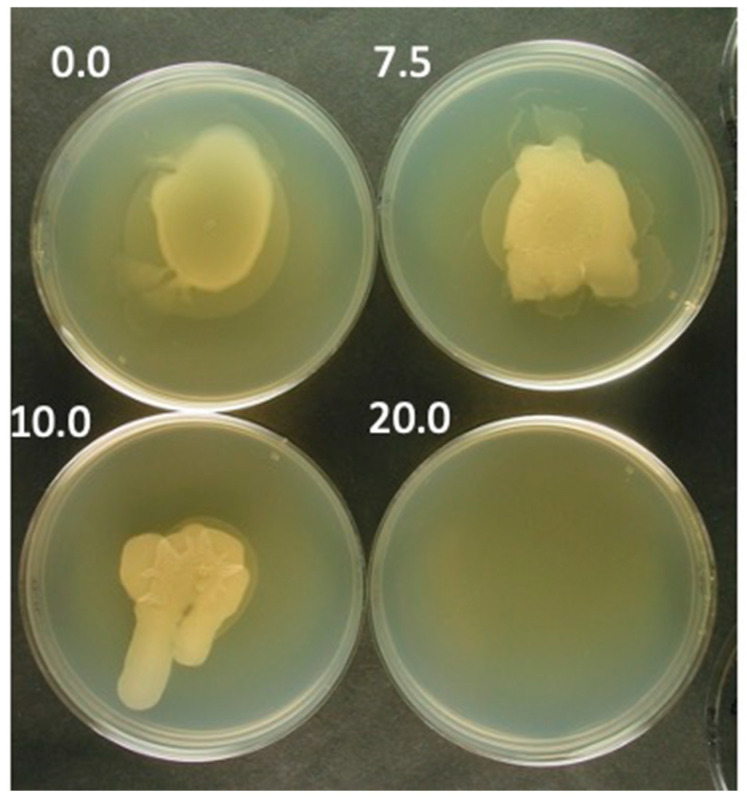
Effect of ATP at uniform concentration. The experiment of Figure 1 was performed with incubation with both segments of the lower agar gel having the same [ATP] so that the [ATP] in the upper agarose gel was uniform. The [ATP] (mM) is indicated in the figure.

As seen in Figure 1, a relatively clear region at the high [ATP] side of a Petri dish increases in size as the [ATP] increases from 0.2 to 10 mM. Without added ATP, the result is in Figure 2, 0.0 dish. When 10 mM ATP is reached, the turbidity spreading is dramatically unilateral, the low [ATP] side being the more turbid. This bias in spreading continues at 20 and 30 mM ATP. At 40 mM ATP, both the bias and the total turbidity are relatively low. Nonetheless, the bacterial turbidity in the region of inoculation is not decreased at 40 mM ATP. This is evidence that the decreased spreading was primarily the result of inhibition of bacterial motility, although inhibition of bacterial propagation was also possible. Previous studies have shown that chelation of iron by ATP is a source of the inhibition of propagation [21].

### 3.2. Effects of ATP at Uniform Concentration

The effects of uniform [ATP] were determined by repeating the experiment in Figure 1, with the following change. The same [ATP] was in both bottom-layer wedges. The results were the following. (1) The spreading was significantly reduced at ATP = 0 (Figure 2; [ATP] in mM is at the upper left of each panel). (2) The results at either 7.5 mM or 10.0 mM ATP were not significantly different (Figure 2). Bias in the turbidity was not observed, although eccentricities, not understood, did occur at all [ATP]s in Figure 2. However, the eccentricities were not systematic and varied among different trials. In contrast, at 20 mM ATP (Figure 2), propagation was reproducibly inhibited to the point that no light scattering was observed. Thus, ATP inhibition of propagation does occur.

However, the unilateral turbidity with 10 mM ATP in the lower agar wedge only (Figure 1) cannot be explained by this inhibition alone in that the inhibition is not detected when 10 mM ATP was in both lower agar wedges in Figure 2. This asymmetry implies preferential motion down artificially generated [ATP] gradients, i.e., ATP is chemorepellent to motile *B. thuringiensis*.

### 3.3. Location of Infective Phage 0105phi7-2 in the [ATP] Gradient

Testing of the above hypothesis, part (2) (i.e., phage follow host cells down an ATP concentration gradient in a non-infective state), was performed by use of the following perturbation of the experiment in Figure 1, with lower wedge [ATP] = 20 mM. After inoculation of bacteria in the center of the Petri dish (origin), incubation was performed for only 3 h before ~165 infective phages were inoculated in the same place as the bacteria. At this point, a second 0.175% agarose overlay was poured. This overlay had bacteria at lawn-producing concentration and was gelled on top of the first 0.175% agarose layer. Then, the Petri dish was incubated for an additional 16 h.

**Figure 3:**
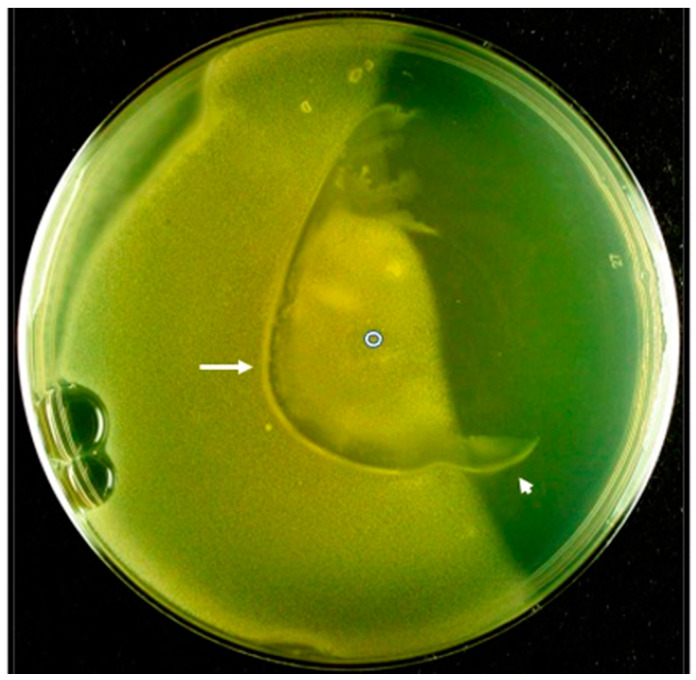
Response of phage 0105phi7-2 to the migration of its host in an [ATP] gradient. The experiment of Figure 1 was repeated with 20 mM ATP in the lower agar wedge (not in the other wedge) and subsequent phage inoculation, followed by addition of a bacterial lawn-producing layer. The high [ATP] side is at the right. The following imperfections are at the left: lower, bubbles; upper, incomplete coverage of lower agar gel.

The result included low [ATP]-biased bacterial turbidity, as expected (Figure 3). The lytic activity of the phages was detected via clearing of the uppermost, bacterial lawn-generating layer. This clearing occurred in an arc (arrow in Figure 3). Thus, chemorepellent activity was confirmed, together with confirmation that at least some phages did follow the 0-time-inoculated host cells down the ATP concentration gradient from the origin. We observed no evidence of phage activity trailing toward the inoculation point (empty circle in Figure 3) from the arc of lysis indicated by the arrow in Figure 3. This observation might be, at least partially, a product of conversion of cells to phage resistance between the point of inoculation and the clear ring (central region). However, the existence of the clear ring implies that the level of both spontaneously resistant cells and lysogenized cells (mostly those in the upper agarose layer) was low enough so that reduction in turbidity of the central region would have occurred if presence of resistant cells was the only mechanism for turbidity in the central region. This reduction was not observed. Thus, non-infectivity of ride-hitching phages is at least part of the explanation for this turbidity. Movement of the phages had occurred with the phages in a relatively non-infective state.

Close observation of Figure 3 reveals the following details that appear non-explainable based on the artificially introduced [ATP] gradient. (1) The arc of phage lysis-induced clearing has a turbid lining selectively on the inoculation point-distal side of the arc (arrow in Figure 3). (2) The arc extends to a region on the high [ATP] side of the position of inoculation (arrowhead in Figure 3). These details are likely explained by the release of a chemotaxis-inducing agent by cells phage lysed during incubation. This agent is likely to be ATP, known to be released by Gram-positive bacteria [22] (review [23]). When data in [22] are translated to the current bacterial concentrations, which are over 10^9^/mL, eATP is in the 1–10 mM range, comparable to concentrations expected in the artificially induced [ATP] gradient.

Qualitatively, the result in Figure 3 was obtained in 10 repetitions of this experiment. The repetitions tested (1) times of phage inoculation between 1 and 3 h, in comparison to the 2 h in Figure 3, and (2) 300, rather than 165, phages inoculated. An arc of clearing was present in all these trials, with no trailing clearing. The arc varied in size and shape but, otherwise, reproduced the pattern in Figure 3, except for the (not understood) widening of the arc of clearing at the top of Figure 3.

## 4. Discussion

The experiments reported here confirm key predictions of the ride-hitching hypothesis above. An alternative hypothesis is that Figure 3-like, directed phage movement is the product of ATP-fueled, bacteria-independent phage motion (i.e., phage swimming). Phage swimming is a possibility considered in other communications and found unlikely, based on current data [15] (review [16]). The observation in Figure 1 of ATP-avoiding (chemorepellent) bacterial motility provides the basis for phage ride-hitching. This is, to our knowledge, the first observation of motility of this type for bacteria.

Details of the ride-hitching remain to be determined. These details include mechanisms of (1) non-infective phage binding, which was previously proposed [15] to be an observed head-host cell binding, and (2) low [ATP]-induced conversion to infective phage binding, which was previously proposed [15] to be the conversion of head-host to tail-host binding.

The following are implications of ride-hitching for phage therapy of bacterial disease. First, a ride-hitching-capable phage can realistically be assumed useful for in-biofilm phage embedding, Trojan-horse-like. Second, a way to encourage both this embedding and, then, bacteriolytic activity is (1) to add ATP distal to the site where bacteriolytic activity is needed and (2) enzymatically lower [ATP] near this site. Effects of this type would be in addition to the anti-bacterial and antibiotic potentiating effect [21,24] of ATP.

To test and, if successful, use this strategy, the phage involved should be lytic [25,26]. However, the genome of phage 0105phi7-2 has genes typical of a lysogenic phage [15]. Presumably, phage 0105phi7-2 has lysogenic ancestors and is likely to still be lysogenic [15]. Thus, (1) the system presented here is a model, and (2) search for ride-hitching, lytic phages is a high priority for the future. To rapidly screen for these phages, one might use the procedure of Figure 3.

The experiments reported here are conducted with simple, inexpensive technology, and they are easily reproducible with additional phage/host combinations. The newest technical feature is the use of highly purified, high-gel-strength agarose.

## Data Availability

The data presented in this study are all available within one or more of the following: Figure and Text.
